# Prevalence and risk factors for common respiratory pathogens within a cohort of pet cats in the UK

**DOI:** 10.1111/jsap.13623

**Published:** 2023-05-29

**Authors:** I. Chan, A. Dowsey, P. Lait, S. Tasker, E. Blackwell, C. R. Helps, E. N. Barker

**Affiliations:** ^1^ Internal Medicine Small Animal Specialist Hospital Sydney New South Wales Australia; ^2^ Bristol Veterinary School University of Bristol Langford UK; ^3^ The Linnaeus Medical Team Linnaeus Group Solihull UK; ^4^ Langford Vets University of Bristol Langford UK

## Abstract

**Objectives:**

Feline herpesvirus (FHV), feline calicivirus (FCV) and *Chlamydia felis* are common causes of upper respiratory tract disease (URTD) in cats. Their prevalence in the UK pet cat population has not been reported and little is known regarding the risk factors for their oral carriage.

**Methods:**

Total nucleic acid was extracted from owner‐collected buccal swabs (n=600) from cats enrolled in a self‐selected longitudinal cohort study. Duplex quantitative PCRs for the detection of FHV and *C. felis* genomic DNA and reverse‐transcriptase quantitative PCRs for the detection of FCV genomic RNA were performed. Duplicates, swabs with insufficient host DNA/RNA, and cats with missing data were excluded. Selected epidemiological data were interrogated using univariable and multi‐variable logistic regression modelling to identify risk factors.

**Results:**

Data from 430 cats were included in the final statistical model. Of these, 2.1% (n=9/430; 95% CI 1.0% to 3.9%) were positive for FHV, 13.3% (n=57/430; 95% CI 10.2% to 16.8%) positive for FCV and 1.2% (n=5/430; 95% CI 0.4% to 2.7%) positive for *C. felis*. FCV co‐infection was present in five (44%) FHV‐positive cats and three (60%) *C. felis*‐positive cats. FCV carriage was more frequent in purebred cats (odds ratio 2.48; 95% CI 1.37 to 4.49) and in cats with current or historical clinical signs compatible with URTD (odds ratio 2.98; 95% CI 1.22 to 7.27).

**Clinical Significance:**

FCV was the most frequently encountered URTD pathogen in this sample of cats; this should be noted for disinfectant choice. In cats suspected of having FHV or *C. felis* infection, assessment for co‐infection with FCV is recommended.

## INTRODUCTION

Feline herpesvirus (FHV), feline calicivirus (FCV) and *Chlamydia felis* are common oral infectious agents of cats, and the main causative agents of feline infectious upper respiratory tract disease (URTD) (Gruffydd‐Jones *et al*. 2009, Radford *et al*. 2009, Thiry *et al*. 2009, Möstl *et al*. 2015). Despite the availability of vaccines against these agents, they continue to be the cause of significant disease to cats (Harbour *et al*. 1991, Radford *et al*. 2006, Ohe *et al*. 2007). Currently, the most sensitive method of detection of these agents is by PCR analysis of nucleic acid (DNA/RNA) purified from swabs collected from sites of viral shedding (*e.g*. buccal, pharyngeal and ocular mucosa) (Reubel *et al*. 1993, Veir *et al*. 2008, Abd‐Eldaim *et al*. 2009, Schulz *et al*. 2015).

In a convenience study of UK cats (Binns *et al*. 2000) using viral isolation on oropharyngeal swabs to determine the presence of FHV and FCV, prevalence was 11% and 33%, respectively, when URTD was reported, and 1% and 21%, respectively, in cats without URTD. A more recent study (Helps *et al*. 2005) using PCR to detect presence of FHV, FCV and *C. felis* in cats from large multi‐cat (>5 cats) households across Europe from oropharyngeal and ocular swabs found prevalences of 16%, 47% and 10%, respectively, where URTD was reported, and 8%, 29% and 3%, respectively, where no clinical signs were reported. The prevalence of *C. felis* differed across studies, ranging from 0% to 32% in cats with URTD, as assessed by a variety of diagnostic tests (using immunofluorescence assays, PCR and isolation) across several countries (Studdert & Martin 1970, Povey & Johnson 1971, Studdert *et al*. 1981, Wills *et al*. 1988, Sykes *et al*. 1999, Rampazzo *et al*. 2003, Fernandez *et al*. 2017), and up to 4.2% (Helps *et al*. 2005, Low *et al*. 2007, Halánová *et al*. 2019) in conjunctival swabs, as assessed by PCR, from cats without clinical signs. PCR‐based prevalence data for FHV, FCV and *C. felis* carriage in pet cats from UK households are limited.

The aim of this study was to determine the prevalence of FHV, FCV and *C. felis* within a population of predominantly young, client‐owned cats in the UK enrolled in a longitudinal cohort study using PCR. A secondary objective was to use prospective data from questionnaires completed by the cats’ owners to determine what risk factors were present that could predict the presence of pathogen carriage.

## MATERIAL AND METHODS

### Population

The Bristol Cats study is a longitudinal study of UK cat health, welfare and behaviour started in Spring 2010 and run by veterinary surgeons, behaviourists and epidemiologists at Bristol Veterinary School, University of Bristol. A number of research papers using this feline cohort have previously been published (Welsh *et al*. 2013, Longstaff *et al*. 2017, Wilson *et al*. 2017, Maniaki *et al*. 2021). Epidemiological data and buccal swabs were prospectively collected at specific timepoints for cats registered to the Bristol Cats study. These were used within this study to assess for the presence of selected URTD infectious agents and the risk factors associated with their carriage.

Cat owners could register 2 to 4‐month‐old kittens for inclusion in the longitudinal study between 2010 and 2016. Owners completed periodic questionnaires regarding cat and owner demographics, cat behaviour, husbandry, clinical signs of disease and veterinary treatment. Questionnaires were sent to owners at specific intervals during their cat's life, including questionnaire one (Q1) at 2 to 4 months; questionnaire two (Q2) at 6 months; questionnaire three (Q3) at 12 months; and questionnaire four (Q4) at 18 months. Owner‐reported observation of clinical signs supports investigation of problems that are either not presented to veterinarians for investigation, or only presented once the clinical signs become more chronic or more serious in nature; hence, data from this study can be used to identify factors with the potential to provide early intervention of problems. Questionnaires were constructed by the Bristol Cats team, which includes specialist veterinary surgeons, behaviourists and epidemiologists.

### Owner‐collected buccal swab samples

From Spring 2012 to Autumn 2016, when the cats were mostly aged between 6 and 18 months, cotton‐tipped swabs, return envelopes and written instructions that included a link to video instructions (see Data S1) were posted to all owners of cats recruited into the Bristol Cats study. Buccal swab samples were collected from the cats by their owners at home. Following their return, the buccal swab samples were stored at −20°C pending analysis. A pseudorandom selection of returned buccal swab samples (n=600) were selected based upon accessibility in storage; this number was used based on the ability for it to detect at least one positive result with a 95% confidence interval (CI) were the true prevalence ≥0.5% (nQuery Advisor, Statsols).

Total nucleic acid (DNA and RNA) was extracted using a high throughput automated platform (Chemagic 360 robot in combination with Chemagic body fluids NA kit, Perkin‐Elmer) in 2019, as previously described (Barker *et al*. 2017). Quantitative PCR assays were run on a high throughput platform (qTower 96, Analytik Jena) using assays that have been previously described; these comprise a duplex quantitative PCR for the detection of FHV and *C. felis* genomic DNA and two reverse‐transcriptase (RT) quantitative PCRs for the detection of FCV genomic RNA (Helps *et al*. 2005). Each assay included an internal amplification control for the presence of feline RNA/DNA. Both negative (RNase‐free water) and positive (pathogen and feline nucleic acid) PCR controls were included in each 96 well plate.

### Review of Bristol cats questionnaire data

As most buccal swabs were collected when the cats were aged between 6 and 18 months, epidemiological data from the owner‐completed questionnaires (Q1 to Q4) pertaining to this period were combined for analysis, as shown in Table 1. Anonymised data pertaining to signalment (breed, sex and neuter status), environment (indoor/outdoor access; presence of other cats within the household) and health (vaccination status; historical or current URTD signs; owner‐reported diagnosis of historical or current “cat flu”) were obtained. These eight potential explanatory variables were selected based on previous studies investigating risk factors for feline URTD (Sykes *et al*. 1999, Fernandez *et al*. 2017).

**Table 1:** Variables assessed as potential risk factors for oral carriage of feline herpesvirus, feline calicivirus and *Chlamydia felis*

Variable	Description	Categories
Breed (Q1)	Breed of the cat	Crossbred (comprising domestic shorthair, domestic longhair and other crossbreeds)
		Purebred (as given by owner; not a “domestic” breed or cross‐breed)
Sex (Q1)	Sex of the cat	Male
		Female
Neuter status (Q3)	Whether the cat was neutered between two‐18 months	Entire
		Neutered
Outdoor access (Q2) (Q3) (Q4)	What indoor and outdoor access the cat was given	Q2
		Indoor only
	For the purpose of univariable analysis, any cats that are not kept exclusively indoors are categorised as having outdoor access	
		Indoor and outdoor access
		Outdoor only
		Q3 and Q4^a^
		Inside only – cat is not allowed outside
		Inside – cat goes out into enclosed “run” or on a lead
		Inside and outside
		Outside only – cat is not allowed in the house
Single or multi‐cat household (Q1) (Q3) (Q4)	Derived from combining the response in Q1, Q3 and Q4	Consistently a single cat household
		Multi‐cat household at any time point
Vaccination (Q2)	Whether the cat has received first vaccination course	Vaccinated
		Unvaccinated
URTD signs observed by owners (Q2) (Q3) (Q4)	Derived from combining the response in Q2, Q3 and Q4	Attacks of wheezing with whistling on the chest
		Attacks of breathlessness
		Sneezing
		Cough
		Runny nose
		Mouth ulcers
		Drooling
		Sore/ red/ runny eyes
Diagnosis of “cat flu” (Q2) (Q3) (Q4)	Derived from combining the response in Q2, Q3 and Q4	Yes – diagnosed by vet
		Yes – but was not seen by a vet
	Whether the cat had been diagnosed with “cat flu” at any time point (“Has your cat had cat flu since you have owned him/her?”)	No

^a^ Additional rows included in “Categories” for outdoor access due to changes in the terminology between questionnaires

### Statistical analysis

Participating cats were identified using their unique Bristol Cats study identification number. Data were entered into a database (Excel for Mac, v16.61, Microsoft) before being transferred into a statistical software package for analysis (SPSS Statistics, v28.0.1.1, IBM), including the descriptive statistics pertaining to demographic and health data. Cats were categorised as “crossbred” if reported by the owner to be Domestic Shorthair/Longhair or cross‐breed (a.k.a. “moggies”) and as “purebred” if reported by the owner to be a specific breed other than the Domestic breeds or crosses. The dependent variables used were the presence or absence of FHV DNA, FCV RNA and *C. felis* DNA. Initially, univariable analysis was conducted using binomial logistic regression to compare detection of pathogen with eight potential risk factors (Table 1).

Due to the low number of cats infected with FHV and *C. felis*, multi‐variable statistical analysis of risk factors was not performed. For FCV infection, any factor with a P‐value ≤0.2 from the univariable analysis was taken forward into building a multi‐variable model using binomial logistic regression as previously described (Maniaki *et al*. 2021). Correlation between independent variables was assessed by multi‐collinearity (tolerance values >0.1). For each dependent variable, cats with missing data for any of the potential risk factors with a P‐value of ≤0.2 were excluded from subsequent analysis. A multi‐variable model was built using a backward elimination process (likelihood ratio test) with the selection of independent variables for removal based on minimising the log‐likelihood‐ratio statistic, as previously described (Maniaki *et al*. 2021). The final model was assessed for fit using Nagelkerke R^2^.

All variables with a P‐value of ≤0.05 were considered statistically significant.

## RESULTS

### Descriptive statistics for the Bristol cats cohort

Overall, 2456 cats recruited into the Bristol cats study. From these, 819 buccal swabs were returned. These were associated with 749 cats (30.5%), with two swabs collected on separate dates for 49 cats (6.5% of cats that returned swabs), and cat identification number not recorded for 21 swabs (2.6% of swabs returned).

#### Quantitative PCR and RT‐PCR assays

**FIG. 1:**
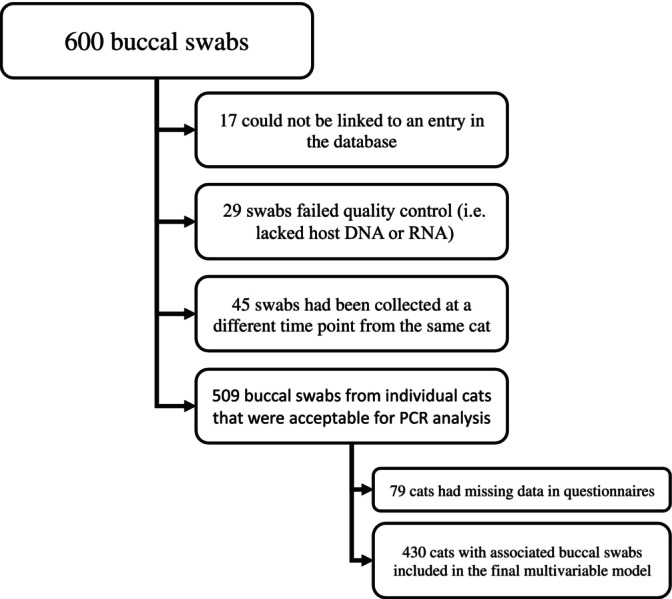
Flow chart showing the number of buccal swabs and reason of case exclusion

Of the 600 swabs selected, 17 could not be associated with an identifiable cat in the database (see Fig. 1). Of the 583 buccal swabs that were associated with an identifiable cat, 90 were duplicate swabs collected from the same cats (*i.e*. two swabs each from 45 cats). Of the 90 duplicate swabs, six were collected at the same time from three cats, all were positive for feline DNA and RNA in both assays, and none were positive for pathogens. Of the 84 duplicate swabs collected at different times from 42 cats, 3 were negative for DNA and RNA in both assays and 6 were negative for RNA in the FCV RT‐PCR (3 were the swab collected first, 3 were the swab collected second), all were from different cats. Two cats were positive for *C. felis*, both on the swab collected first. One cat was positive for FHV, from the sample collected first. Twelve of the cats were positive for FCV, three on both swabs, four on the first collected swab, five on the second collected swab; one of these was concurrently positive for *C. felis* and another concurrently positive for FHV. In addition, 29 failed quality control with neither feline DNA nor feline RNA being amplifiable (no feline DNA or RNA were detected in 10); feline DNA alone was not detected in a further five, and feline RNA alone was not detected in a further 14. In total, 91 swabs were excluded from further evaluation, leaving 509 for analysis.

#### Demographic data

Seventy‐nine additional cases of 509 were excluded due to missing data in the questionnaires – out of 509 cats, 15 had unknown breed status, 11 had unknown sex, 26 had unknown neuter status, 24 had unknown previous diagnosis of “cat flu” and a further three had unknown number of cats in the household, leaving 430 cats for the multi‐variable analysis (Fig. 1). The demographic data of the individual cats can be found in Table 2.

**Table 2:** Owner reported data from various time points for cats included in the study (n=430)

Signalment			
Purebred	326 (75.8%) were crossbred		
	271 domestic shorthair (a.k.a. shorthair “moggy” or cross‐breed)		
	43 domestic longhair (a.k.a. longhair “moggy” or cross‐breed)		
	104 (24.2%) were purebred		
	Comprising 25 British shorthair, 17 Maine Coon, 12 Siamese, 3 Tonkinese, 3 Siberian, 3 Oriental, 2 Persian, 2 Bengal, 2 Burmese, 2 Ragdoll, 2 Devon Rex, 2 Birman, 2 Norwegian Forest, 2 Korat, 2 Russian Blue, 1 Selkirk Rex, 1 Scottish Fold, 1 Ragamuffin and 20 “other” breeds		
Sex	246 (57.2%) were male		
	184 (42.8%) were female		
Environment			
Additional cats in the household	At time of acquisition (Q1), 44 (10.2%) were singletons and 386 (89.8%) lived with one or more cat	At 12 months of age (Q3), 40 (9.3%) were singletons and 390 (90.7%) lived with one or more cat	At 18 months of age (Q4), 76 (17.7%) were singletons, 333 (77.4%) lived with one or more cat and 21 (4.9%) were not recorded/ unknown
Outdoor access	At 6 months of age (Q2), 146 (34.0%) were kept exclusively indoor, 249 (57.9%) were considered indoor/outdoor, 23 (5.3%) were kept exclusively outdoors and 12 (2.8%) were not recorded / unknown	At 12 months of age (Q3), 27 (6.3%) were kept exclusively indoor, 204 (47.4%) were considered indoor/outdoor, 109 (25.3%) were kept exclusively outdoors and 90 (20.9%) were not recorded / unknown	At 18 months of age (Q4), 7 (1.6%) were kept exclusively indoor, 317 (73.7%) were considered indoor/outdoor, 11 (2.6%) were kept exclusively outdoors and 95 (22.1%) were not recorded / unknown
Health/Veterinary Care			
Vaccination	At 6 months of age (Q2)		
	399/430 (92.8%) were vaccinated		
	17/430 (4.0%) were unvaccinated		
	14/430 (3.3%) were not recorded/unknown		
Neuter	By around 1 year of age (Q3)^a^		
	412 (95.8%) were neutered		
	18 (4.2%) were entire		
Diagnosis of “cat flu”	At 6 months of age (Q2), 382 (88.8%) had not been diagnosed with “cat flu,” 16 (3.7%) had been diagnosed with “cat flu” and 32 (7.4%) were not and recorded/unknown	At 12 months of age (Q3), 401 (93.3%) had not been diagnosed with “cat flu,” 20 (4.7%) had been diagnosed with “cat flu” and 9 (2.1%) were not recorded / unknown	At 18 months of age (Q4), 407 (94.7%) had not been diagnosed with “cat flu,” 19 (4.4%) had been diagnosed with “cat flu” and 4 (0.9%) were not recorded / unknown
Upper respiratory tract signs	At 18 months of age (Q4)		
	411 (95.6%) had no upper respiratory tract signs reported		
	19 (4.4%) had one or more upper respiratory tract sign reported (including nasal discharge; sneezing; cough; ptyalism; oral ulcers; ocular inflammation)		

^a^ The ratio of neutered *versus* entire cats did not change over the subsequent 6 months, although fewer cats had their neuter status reported at Q4

**FIG. 2:**
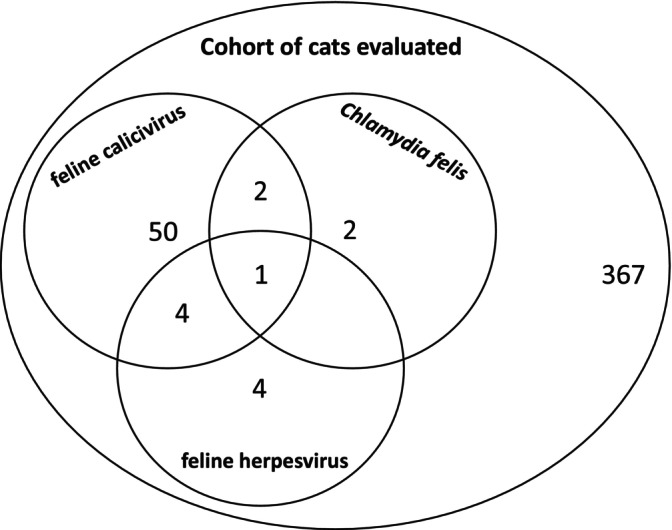
Venn diagram illustrating the distribution of selected pathogen detection in this cohort of pet cats

Of the 430 cats included in the study, 9 (2.1%; 95% CI 1.0% to 3.9%) were positive for FHV, of which five were also positive for FCV, including one cat that was also positive for *C. felis* (Fig. 2). Of these FHV‐positive cats, four were domestic long/shorthairs, two were British shorthairs and one each of three other breeds. Fifty‐seven of the 430 (13.3%; 95% CI 10.2% to 16.8%) cats were positive for FCV, of which five were also positive for FHV, including one cat that was also positive for *C. felis*. Of the 57 FCV positive cats, 34 were crossbred, 23 were purebred (comprising 7 Maine Coon, 6 Siamese, 2 Orientals, 2 were recorded as unknown and 1 each of 6 other breeds). Five of 430 cats (1.2%; 95% CI 0.4% to 2.7%) were positive for *C. felis*, of which 3 were also positive for FCV, including one cat that was also positive for FHV (Table 3). All were domestic shorthairs.

**Table 3:** Overall prevalence of pathogens detected

	Overall	Co‐infection
FHV	2.1% (n=9/430; 95% CI 1.0% to 3.9%)	55.6% (n=5/9)
FCV	13.3% (n=57/430; 95% CI 10.2% to 16.8%)	12.3% (n=7/57)
*C. felis*	1.2% (n=5/430; 95% CI 0.4% to 2.7%)	60.0% (n=3/5)

Abbreviations: *C. felis*, *Chlamydia felis*; CI, confidence interval; FCV, feline calicivirus; FHV, feline herpesvirus

#### Univariable logistic regression analysis

The dependent variables used were the presence or absence of FHV DNA, FCV RNA and *C. felis* DNA. Eight potential predictor variables for pathogen carriage were considered for univariable analysis with results shown in Table 4. Being a purebred was a risk factor for FCV (P<0.001; odds ratio 2.71, 95% CI 1.60 to 4.59). Being entire at 12 months was a risk factor for both FCV (P=0.008; odds ratio 3.67, 95% CI 1.41 to 9.57) and *C. felis* (P=0.005; odds ratio 12.69, 95% CI 2.18 to 73.90). Living in a multi‐cat household was associated with a high risk of FCV (P=0.033; odds ratio 8.81, 95% CI 1.20 to 64.84). A previous diagnosis of “cat flu” was associated with oral carriage of both FCV (P=0.030; odds ratio 1.58, 95% CI 1.04 to 2.40) and *C. felis* (P=0.017; odds ratio 2.97, 95% CI 1.22 to 7.24). The presence of URTD signs was a risk factor for *C. felis* (P=0.038; odds ratio 4.24, 95% CI 0.49 to 36.96).

**Table 4:** Predictor variables considered for univariable analysis of risk factors for oral carriage of feline herpesvirus (FHV), feline calicivirus (FCV) or *Chlamydia felis* (*C. felis*). Significant results are in bold

Pathogen	FHV			FCV			*C. felis*		
	Positive cats (n)	P‐value	OR (95% CI)	Positive cats (n)	P‐value	OR (95% CI)	Positive cats (n)	P‐value	OR (95% CI)
Purebred									
Crossbred	4	0.151	1.0	39	**<0.001**	1.0	7	0.996	N/A
Purebred	5		2.1 (0.76 to 5.78)	30		2.71 (1.60 to 4.59)	0		
Sex									
Male	5	0.917	1.0	33	0.067	1.0	2	0.252	1.0
Female	4		1.07 (0.29 to 4.06)	37		1.608 (0.97 to 2.67)	4		2.71 (0.49 to 14.94)
Neuter status (Q3)									
Neutered	1	0.315	1.0	59	**0.008**	1.0	4	**0.005**	1.0
Entire	8		2.98 (0.36 to 25.05)	7		3.67 (1.41 to 9.57)	2		12.69 (2.18 to 73.90)
Multi‐cat (Q3)									
Single cat household	0	0.998	N/A	1	**0.033**	1.0	0	0.998	N/A
Multi‐cat household	9			68		8.81 (1.20 to 64.84)	6		
Vaccination (Q2)									
Vaccinated	9	0.999	N/A	64	0.368	0.59 (0.19 to 1.85)	6	0.189	0.23 (0.03 to 2.05)
Unvaccinated	0			4		1.0	1		1.0
Outdoor access									
Indoor only	0	0.999	N/A	2	0.800	1.0	0	0.608	0.561 (0.062 to 5.100)
Outdoor access	9			53		1.10 (0.53 to 2.28)	6		1.0
Clinical signs									
Cat flu diagnosed	4	0.163	1.0	11	**0.030**	1.58 (1.04 to 2.40)	3	**0.017**	2.97 (1.22 to 7.24)
Cat flu not diagnosed	5		0.92 (0.77 to 4.78)	54		1.0	3		1.0
URTD signs absent	6	0.093	1.0	64	0.196	1.0	6	**0.038**	1.0
URTD signs present	3		14.21 (3.27 to 61.65)	7		3.58 (1.38 to 9.30)	1		4.24 (0.49 to 36.96)

*Note*: Clinical signs included owner‐reported attacks of wheezing with whistling on the chest, attacks of breathlessness, sneezing, cough, runny nose, mouth ulcers, drooling and sore/ red/ runny eyes.

Abbreviations: CI, confidence interval; OR, odds ratio; URTD, upper respiratory tract disease

#### Multi‐variable logistic regression analysis for factors associated with FCV infection

Six potential risk factors of FCV infection, each with univariable P‐values less than 0.2, were further analysed in a multi‐variable model (Table 5). There was no evidence of multi‐collinearity, and so all were included in the multi‐variable analysis. A backward stepwise regression was performed to ascertain the effects of purebred status, sex, neuter status (at 12 months), the presence of other cats in the household (at 12 months), previous diagnosis of “cat flu” and the presence of current or historic clinical signs associated with URTD on the likelihood that the cats are carriers of FCV. The logistic regression model was statistically significant, *χ*2=19.2, P<0.001. Of the six predictor variables, only two were statistically significant: being purebred and having been previously diagnosed with “cat flu.” Purebred cats had 2.48 times higher odds of carrying FCV compared to crossbred cats (95% CI 1.37 to 4.49). Cats with a previous diagnosis of “cat flu” had 2.8 higher odds of carrying FCV (95% CI: 1.22 to 7.27). The final multi‐variable model had a‐2 log likelihood of 317.3, classified 86.7% of cats correctly and had an effect size (as assessed by Nagelkerke R^2^) of 0.080, explaining 8% of the variability in the model.

**Table 5:** Multi‐variable logistic regression modelling steps for the investigation of risk factors for feline calicivirus (FCV) infection in the 430 cats for which a complete data set were available. Factors with a P‐value ≤0.2 from the univariable analysis were taken forward into building the model. Correlation between independent variables was assessed by multi‐collinearity (tolerance values >0.1). The model was built using a backward elimination process (likelihood ratio test) with selection of independent variables for removal based on minimising the log‐likelihood‐ratio statistic

		Standard error	Wald test	Significance	^e^ Exp(B)	95% confidence interval for^e^ Exp(B)	
						Lower	Upper
Step 1	Purebred^a^	0.309	7.804	0.005	2.371	1.294	4.345
	Sex	0.296	1.927	0.165	1.509	0.844	2.698
	Neutered by Q3	0.584	2.289	0.130	2.421	0.770	7.607
	Multi‐cat^b^ at Q3	1.034	3.260	0.071	6.467	0.852	49.052
	Cat flu^c^	0.847	0.982	0.322	2.315	0.440	12.179
	Clinical signs^d^	0.979	0.160	0.689	1.479	0.217	10.064
Step 2	Purebred^a^	0.307	8.111	0.004	2.400	1.314	4.383
	Sex	0.295	1.852	0.174	1.494	0.838	2.665
	Neutered by Q3	0.583	2.182	0.140	2.367	0.755	7.422
	Multi‐cat^b^ at Q3	1.033	3.328	0.068	6.589	0.869	49.948
	Cat flu^d^	0.459	5.927	0.015	3.056	1.243	7.510
Step 3	Purebred^a^	0.306	7.975	0.005	2.372	1.302	4.319
	Neutered by Q3	0.575	2.715	0.099	2.578	0.836	7.949
	Multi‐cat^b^ at Q3	1.033	3.260	0.071	6.460	0.853	48.945
	Cat flu^d^	0.456	5.855	0.016	3.015	1.233	7.369
Step 4	Purebred^a^	0.303	8.984	0.003	2.480	1.369	4.491
	Multi‐cat^b^ at Q3	1.030	3.064	0.080	6.069	0.806	45.713
	Cat flu^d^	0.455	5.753	0.016	2.979	1.221	7.268

*Note*: Significance shows the P‐value associated with each predictor variable; Q3, questionnaire three (filed at 12 months age)

^a^ Cats were categorised as “crossbred” if reported by the owner to be domestic shorthair/longhair or cross‐breed and as “purebred” if reported by the owner to be a specific breed other than the domestic breeds or crosses

^b^ Multi‐cat, living with one or more other cat

^c^ Cat Flu, current or historical diagnosis of cat flu with or without veterinary confirmation

^d^ Clinical signs, current or historical presence of clinical signs supportive of upper respiratory tract disease

^e^ Exp(B), exponential of unstandardised beta coefficients

## DISCUSSION

All three major upper respiratory tract pathogens, FHV, FCV and *C. felis*, were detected in our population of client‐owned cats. The prevalence of each pathogen was lower than in previous studies (Binns *et al*. 2000, Helps *et al*. 2005) that used either cell culture or PCR, although the relative proportion of FCV‐positive cats was greater when compared with cats infected with either FHV or *C. felis*. The overall lower prevalence of each pathogen could be a true reflection of this cohort of cats from which the samples were derived (*i.e*. predominantly junior life‐stage general pet cat population) as compared to the earlier studies that exclusively or predominantly drew from multi‐cat populations with a bias towards households containing cat(s) with URTD disease, rescue shelters and breeding catteries that likely included a greater representation of older cats. Alternatively, the lower prevalence in this study could reflect sample collection techniques, *i.e*. an inexperienced owner may be less likely to collect a representative sample than a veterinary surgeon; or sample sites, *i.e*. it may be less likely to collect a representative sample from buccal swabs *versus* oropharyngeal swabs combined with nasal or ocular swabs (Veir *et al*. 2008, Schulz *et al*. 2015). The proportional increase in FCV prevalence in this cohort could be due to the buccal swabs being more sensitive in detecting FCV than FHV and *C. felis* given the pathogen predilection sites (Sykes *et al*. 2001, Marsilio *et al*. 2005, Schulz *et al*. 2015) in combination with pathogen load (*i.e*. if cats infected with FCV shed virus at significantly higher copy numbers, then detection will be possible in samples of lower quality where other pathogens might not be detected), or it could reflect the challenge of the PCR primer design due to the FCV antigenic drift. Furthermore, since recrudescence of latent FHV infection usually occurs following periods of stress (Pedersen *et al*. 2003, Thiry *et al*. 2009), swab samples collected in a home environment may detect a lower proportion of FHV carriers due to reduced stress compared to samples collected in veterinary practices. To maximise sensitivity for pathogen detection, particularly in the clinical setting, it is recommended that samples be collected from multiple sites and combined for analysis (Veir *et al*. 2008; Schulz *et al*. 2015). However, owner‐collected buccal samples have been shown to be an acceptable alternative to veterinarian‐collected samples for epidemiological research (Möbius *et al*. 2013), especially when given written and video instructions (as in this case), while even with experience, collection of adequate nasal swab samples has proven more difficult (due to time consumption and cat discomfort) (Veir *et al*. 2008) and can result in owners failure to return samples or indicate concern or difficulty in their collection (Möbius *et al*. 2013).

The majority of cats with either FHV or *C. felis* were co‐infected with FCV. While carriage of FCV was associated with being a purebred cat, *C. felis* was only detected in crossbred cats (although the numbers infected were too small to determine the statistical significance of this). This was in alignment with a previous study which reported that crossbred, longhair cats had a lower infection rate of FCV in young cats below year of age (Wardley *et al*. 1974). Although purebred status was not associated with the presence of one or more other cats in the household, cats may acquire persistent infection from breeding catteries early in life before rehoming to their eventual owners. Further investigations to evaluate acquisition source of the cats may be helpful to ascertain the role of breed in predisposing to FCV infection. Unsurprisingly, detection of FCV was more frequent in cats suspected of having had cat flu. It is not possible to establish whether this reflects chronic shedding with the isolate involved with the cat flu or de novo infection with a different viral strain.

Contrary to previous studies (Wardley *et al*. 1974, Radford *et al*. 2009) detection of FCV was not found to be associated with living in a multi‐cat household in multi‐variable analysis. This was likely to be a type II statistical error, considering the mode of transmission of FCV is through close contact with infected cats (Radford *et al*. 2009), and a multi‐cat household was identified as a risk factor in univariable analysis. Alternatively, it is possible that the association was weakened by cats from single‐pet households having outdoor access and contact with other cats in the neighbourhood.

Although vaccines including *C. felis* are available, their regular use in all cats is not recommended and these vaccines are considered non‐core (Gruffydd‐Jones *et al*. 2009, Day *et al*. 2016). Whether or not any of the cats in the study had received one or more vaccines for *C. felis* was not evaluated due to the design of the questionnaires, and because many (n=335/ 430) cats had received their primary vaccination course, which could have included *C. felis* vaccination, before transfer of ownership and capture within the questionnaire data. Vaccination against both FHV and FCV are considered core, indicated in all cats (Day *et al*. 2016); however, no vaccines against these pathogens induce sterilising immunity, such that carriage and, in some cases, disease is still possible. This likely explains the lack of association between vaccination status and pathogen detection. It is also possible that cats had become infected before receiving their first vaccination.

Helps and others (Helps *et al*. 2005) found that cats with URTD were more likely to be carrying one or more upper respiratory tract pathogens. A similar association was found in this study, with cats having an owner reported diagnosis of “cat flu” being more likely to have FCV and *C. felis* detected. However, the presence of one or more clinical signs associated with URTD was not associated with pathogen detection. This could be due to the episodic nature of shedding for some organisms, and the potential for owners to forget historical clinical signs that their cat may manifest.

Cohort studies can assess causality and consequently provide strong scientific support for putative risk factors (Song & Chung 2010); however, studies like this are not without limitations. While the Bristol Cats cohort was generally similar to that reported in a large cross‐sectional UK study, it included a higher proportion of purebred cats than the general UK population (Murray & Gruffydd‐Jones 2012, Wilson *et al*. 2017). Given that this study identified purebred status as a risk factor in pathogen carriage, this could have impacted on the ability to detect other risk factors, since owners of purebred cats may be more likely to enrol in the study and thereby causing a selection bias. Samples were collected by owners that may have been unfamiliar with sample collection techniques, reflected in the absence of detectable DNA in 18 swabs from 15 cats, and the absence of detectable RNA in 33 swabs from 24 cats; however, owner‐collected buccal swabs have been shown to be an adequate and cost‐effective alternative to veterinarian‐collected buccal swabs in epidemiological studies (Möbius *et al*. 2013). Unfortunately, swabs were only available from a proportion of cats recruited into Bristol cats study, making this a convenience study; this could have introduced bias, potentially through a desire of owners of cats with URTD to collect samples aimed at investigation of feline health, through owners of cats with URTD being reluctant or unable to sample from the mouth due to increased concerns regarding or manifestation of oral discomfort, or alternatively an occult shared risk factor between pathogen carriage and sample collection (*e.g*. behaviour, facial conformation and outdoor access). It was not possible to explore the cat and owner factors underpinning samples submission. Although sample storage and preparation errors cannot be excluded, sample preparation and analysis were performed in a large commercial laboratory that processes thousands of samples every year, so this was considered less likely. Detection of pathogens present at only low levels, as is often seen in carrier states, would also have been limited by any suboptimal sample collection technique. It was not possible to only analyse data from the questionnaire completed closest to estimated date of swab collection for a variety of technical reasons; therefore, data from questionnaires 1 to 4 were pooled as shown in Table 1. Consequently, it was not possible to ascertain the chronicity of pathogen carriage, or prevalence of FHV, FCV and *C. felis* specifically when the cats displayed clinical signs of URTD. In addition, completion and submission of questionnaires was entirely voluntary, based on owner recall, and owners could elect to leave sections blank. This was most obviously reflected in fewer cats being recorded as neutered at 18 months than at 12 months. Similarly, owners were asked at both 6 and 18 months whether their cat had ever had cat flu, with some indicated “yes” at 6 months but “no” at 18 months. All of these factors have the potential to increase the risk of type II statistical error. Future studies with a larger sample size, and sequential swabs from the same cats would help to further elucidate the environmental risk factors that may predispose to oral carriage of FHV, FCV and *C. felis*.

Compared with FHV and *C. felis* carriage, FCV was the most frequently encountered pathogen in the population of UK pet cats included in this study, where it remains relatively common. Risk factors associated with oral carriage of FCV include purebred status, in addition to current or historical clinical signs compatible with URTD. When needing to hospitalise cats, selection of disinfectants that have activity against FCV is paramount (Addie *et al*. 2015) and this is particularly the case in cats with a history of URTD. Most cats in this study with either FHV or *C. felis* were co‐infected with FCV. Therefore, in cats suspected of having *C. felis* or FHV infection, assessment for co‐infection with FCV is recommended, as separate specific management may be required.

### Conflict of interest

P. Lait works for the Diagnostic Laboratories, Langford Vets. C. R. Helps and E. N. Barker have previously worked for the Diagnostic Laboratories, Langford Vets. The Diagnostic Laboratories undertakes molecular testing for infectious and genetic diseases in cats.

### Author contributions

**I Chan:** Data curation (equal); formal analysis (lead); investigation (equal); methodology (equal); writing – original draft (lead); writing – review and editing (lead). **A Dowsey:** Formal analysis (equal); methodology (equal); writing – review and editing (equal). **P Lait:** Data curation (equal); investigation (equal); writing – review and editing (equal). **S Tasker:** Conceptualization (lead); funding acquisition (equal); resources (equal); writing – review and editing (equal). **E Blackwell:** Conceptualization (equal); data curation (lead); funding acquisition (equal); resources (equal); writing – review and editing (equal). **CR Helps:** Conceptualization (equal); funding acquisition (equal); methodology (equal); resources (equal); writing – review and editing (equal). **EN Barker:** Conceptualization (equal); data curation (lead); formal analysis (equal); funding acquisition (equal); methodology (lead); resources (lead); supervision (lead); writing – original draft (equal); writing – review and editing (equal).

### Ethical statement

The collection, storage and use of questionnaires and buccal swabs, as part of Bristol Cats study, were approved under ethical review by the University of Bristol Animal Welfare and Ethical Review Board (UIN/12/0606 with annual renewal; VIN/18/007).

## Supporting information


**Data S1.** Supplementary Information.
