# Supplementary material for: Prevalence and risk factors for common respiratory pathogens within a cohort of pet cats in the UK
Source: J Small Anim Pract. 2023 May 29;64(9):552–60. doi: 10.1111/jsap.13623 (PMC10953343; doi:10.1111/jsap.13623)
Supplement: Supplementary file 1 — Data S1. Supplementary Information. [file JSAP-64-552-s001.docx]

**Supplemental document A**

**Instructions given to owners on how to collect buccal swabs (NB: owners were also requested to collect hair samples from their cat at the same time).**

**The cheek cell sample**

The cheek cell sample will enable us to examine DNA from the ‘Bristol Cats’ to investigate possible genetic components to some common feline problems. We have provided you with instructions, but highly recommend that you also watch the instructional video available at <http://www.langfordvets.co.uk/diagnostic-laboratories/diagnostic-laboratories/general-info-breeders/how-swab-your-cat>. [Link no longer active, however video is still available online <https://youtu.be/uByGqUGZRrc>; accessed 13^th^ January 2023]

To take the cheek cell sample:

1. Your cat’s mouth should be free of food therefore delay sampling for an hour after your cat has eaten.
2. Twist the cap to open the container. Try not to touch anything with the tip of the swab before you take the sample.
3. Carefully raise the top lip on one side of your cat’s mouth.
4. Brush the swab gently on the inside of the lip, between the gums and inside of the cheek.
5. Rotate the swab 3 or 4 times, avoiding the teeth and tongue.
6. Remove the swab from your cat’s mouth and place it back in the container.
7. Label the plastic tube with the cat’s name, ID number (if known) and date the sample was taken.

When you have taken both samples, please place the sealed hair sample bag and the swab together in the ‘biohazard’ bag. Please ensure both samples are labelled and return them in the envelope provided within 24 hours of taking the swab.

The returned samples will be stored, and analysed at a later date, once we have enough samples returned by owners.

If you have any questions please do not hesitate to contact us either on the number or email address above.
